# The Diploma Traffic

**Published:** 1885-01

**Authors:** 


### "THE DIPLOMA TRAFFIC."

Under the above heading *The Dental Record,* of London, briefly reviews some of the abuses of authority on the part of American colleges. The editor closes the article by saying :—

"The loose manner in which degrees are conferred by certain dental colleges in the United States is notorious. Justice and good faith to those graduates who have complied with the educational requirements are looked for, and the honor and status of the dental profession demand their careful keeping. The bold course of action taken in this matter by the INDEPENDENT PRACTITIONER deserves the thanks of the profession at large."

That bold course made us enemies, as we expected it would, but the results have been even greater than we anticipated. We can assure our contemporary that we believe a substantial reform has been brought about, that this standing reproach to American dentistry has been removed, and that hereafter all reputable American colleges will, after this present term, refuse to graduate students except after attendance upon at least two full courses of lectures, of not less than five months each, the separate courses to be delivered in different years. We believe that *The Record* will be enabled to amend its expression in the first line quoted, and in future to say only—"The loose manner in which degrees *were* conferred, etc." At any rate, woe be to the college that infringes the new rule. State laws will enable State societies to throw out their diplomas, and prevent their graduates from entering upon practice, and this is a power that the larger States will not hesitate to use.

